# Oroya Fever, Verruga Peruana, and Other Bartonelloses Incidence Rates in Colombia (2009-2013)

**DOI:** 10.7759/cureus.3528

**Published:** 2018-10-31

**Authors:** Liceth Carolina Urrutia, Andrés Mauricio Patiño-Barbosa, Felipe Arroyave-Valencia, Juan Alejandro Sabogal-Roman, Jaime A Cardona-Ospina, Alfonso J. Rodriguez-Morales

**Affiliations:** 1 Epidemiology and Public Health, Universidad Tecnológica De Pereira, Pereira, COL

**Keywords:** vector-borne disease, bartonella, epidemiology, colombia

## Abstract

Background

*Bartonella bacilliformis*, the etiological agent of Carrion's disease and presumed to be transmitted by phlebotomine sandflies, is endemic to the high-altitude valleys of the South American Andes, including Colombia.

Methods

This observational, retrospective study in which the incidence of bartonelloses (International Classification of Diseases, 10th revision (ICD-10) codes A44.0-A44.9) in Colombia, from 2009-2013, was estimated based on data extracted from the personal health records system (*Registro Individual Prestación Servicios*, RIPS). Using the official population estimates of the National Statistics Department (*Departamento Administrativo Nacional de Estadísticas*, DANE), crude and adjusted incidence rates were estimated (cases/100,000 population).

Results

A total of 1,389 cases were reported (median 289/year), for a cumulative national rate of 3.02 cases/100,000 population; 91.2% were female; 66.8% were <40-year-old (3.8% <9.9-year-old). The cases were 2.9% Oroya fever (A44.0), 13.1% verruga peruana (A44.1), and the rest (85.3%) were other forms of bartonelloses (A44.8-A44.9). The highest rates of Oroya fever were reported in Bolivar (2.5 cases/1,000,000 population). For verruga peruana highest number of cases were reported in Antioquia (32; 17.8%; 5.21 cases/1,000,000 population) and the highest rate at Magdalena (11.54 cases/1,000,000 population) (Risaralda, 6.45; Caldas, 5.1). For other forms of bartonelloses, the highest rates were reported at Magdalena (48.65 cases/1,000,000 population), followed by Huila (32.8) and La Guajira (18.9). At Nariño, Putumayo, Amazonas, Cauca, and Valle del Cauca, 11.7% of the cases of the country were reported.

Conclusions

*Lutzomyia columbiana,* the potential vector of *Bartonella bacilliformis* in Colombia, is distributed not only in Nariño, Cauca, and Valle del Cauca but also in the Antioquia, Caldas, Huila, La Guajira, Risaralda, Cordoba, and Caribbean areas. Given this distribution, the transmission would be occurring, as seen in reported cases, in more areas than previously described by classic reports of these diseases in the country.

## Introduction

Bartonelloses are infections caused by several species of bacteria in the *Bartonella* genus. These are gram-negative bacteria and facultative intracellular parasites. Three species of the genus *Bartonella* are known to be important causes of human diseases: *Bartonella* (*B.) henselae*, the etiological agent of the cat-scratch disease, bacillary angiomatosis, and peliosis, infections that are usually benign and self-limited [1-2]; *B. quintana, *which causes trench fever, and *B. bacilliformis*, responsible for Carrion’s disease (or verruga peruana). Other species can be associated with human disease. The infections caused by those species have various epidemiological characteristics, clinical manifestations, and treatment approaches [2-3]. In some countries, particularly in Latin America, infections due to *B. bacilliformis* are endemic and relevant.

*Bartonella bacilliformis*, which uses a polar flagellum for motility, adheres and invades red blood cells, where it replicates in vacuoles. They also synthesize an endothelial cell-stimulating factor, causing proliferation of both endothelial cells and blood vessels, leading to clinical manifestations, bacteremia, and the development of the illness [2-3].

Carrion’s disease, caused by *Bartonella bacilliformis,* is common in some Latin American countries, such as Peru, Ecuador, and Colombia, as well as the Peruvian Andes, as has been widely reported. Some studies have reported seroprevalences as high as 75%. This pathogen has been identified in domestic cats. At the same time, cats are also infected with* B. henselae*. The ecology of those infections, which are vector-borne, as is *Bartonella bacilliformis*, and transmitted by bites from sand flies of the genus *Lutzomyia*, is limited to the altitude niche of 3,000 to 10,000 feet. Infections due to *B. bacilliformis *led to two different clinical diseases: an acute bacteremia illness (called Oroya fever) and an indolent cutaneous eruptive chronic condition (verruga peruana or Peruvian wart) [3-4].

Although several cases are found in the Andes mountains and its endemic areas beyond Peru, other countries, such as Ecuador and Colombia, have not studied such infections in detail. In the case of Colombia, this disease has been particularly reported in the Southern areas, the border with Ecuador and Peru, but, in general, it has been neglected. The Colombian territory has climatic, geographic, economic, and epidemiological conditions adequate for the transmission of this and other bacterial and vector-borne diseases (VBD), such as malaria, Chagas disease, and leishmaniasis. In particular, leishmaniasis is still prevalent and shares with some of these bartonelloses, the same group of vectors, the sand flies. In the case of malaria, large efforts have been relevant in the reduction of the disease, moving towards elimination, but this VBD has been largely studied. This is not the case for bartonelloses [5-8].

The objective of this study was to estimate the incidence of bartonelloses in Colombia, based on an assessment of cases notified to the health information system during the period 2009 to 2013.

## Materials and methods

Colombia is a South American country constituted by 32 departments (main administrative levels), with a total population of 48,747,632 in 2016. The Colombian territory presents climatic, geographic, and epidemiological conditions suitable for the transmission of *Bartonella *and other bacteria and vector-borne pathogens. As in other tropical countries, Colombia consists of large areas where environmental factors, such as temperature, humidity, precipitation, and altitude, as well as socio-economic factors are suitable for transmission.

We performed an observational retrospective study. Epidemiological data were collected from the Individual Health Records System (*Registro Individual de Prestación de Servicios*, RIPS). The International Classification of Diseases 10th revision (ICD-10) codes A44.0-A44.9 were used to obtain the number of cases from each department of Colombia from 2009-2013, given the fact that bartonelloses are not under the surveillance system and this is a passive capture health information system. This information came from confirmed cases and the Colombian National Institute of Health reviewed data quality. Data were obtained with the agreement of the Ministry of Health and Social Protection through the Protection Information System (SISPRO) via a client access server, SISPRO RIPS. Data used for this study were obtained from confirmed cases that were reviewed in terms of data quality, initially from data from the National Institute of Health, Colombia, and afterward by SISPRO and its Data Cubes system. Data for this study came from 33 reference notification units, one per department, and were later consolidated and centralized in Bogotá into the SISPRO system. Currently revised and consolidated data are available for the period 2009–2013. The quality of RIPS data in Colombia has been described elsewhere [9].

Finally, using official reference population data from the National Administrative Department of Statistics (*Departamento Administrativo Nacional de Estadísticas*, DANE), estimates of annual incidence rates for all departments of the country during the study period were calculated (32 departments and the capital district for five years) (cases/1,000,000 population) to provide, for the first time, estimates of bartonelloses incidence in the country by department. Incidence rates were also estimated by age group. In addition, maps of the high incidence departments were developed using the free software Kosmo SIG (SAIG S.L., Seville, Spain), a geographical information system.

## Results

**Figure 1:**
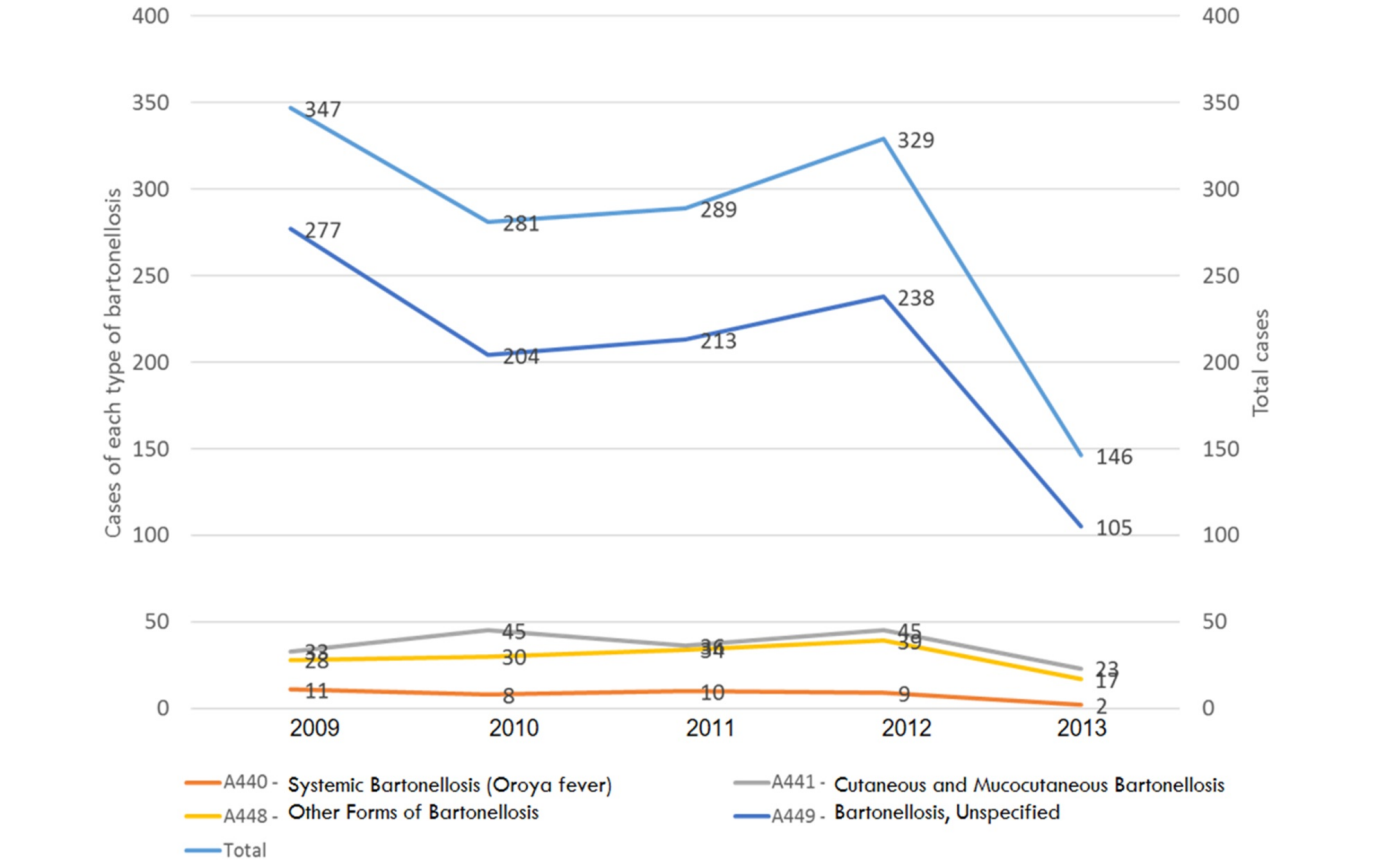
Bartonelloses, Colombia, 2009-2013

During the study period, 1,389 cases were reported (median 289/year), for a cumulative national rate of 3.02 cases/100,000 population, showing a decrease from 347 cases in 2009 to 146 in 2013 (Figure 1).

**Figure 2:**
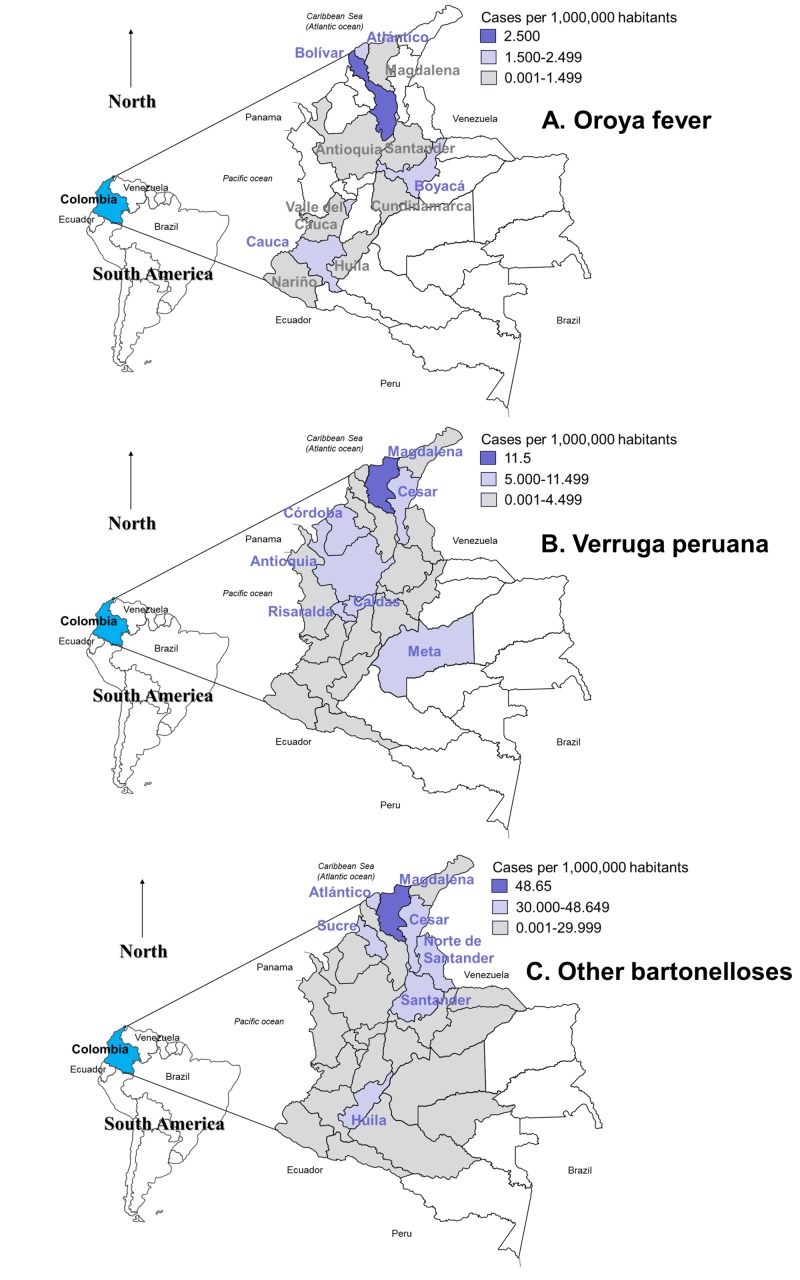
Geographical distribution of bartonelloses in Colombia, 2009-2013 Departments in white have not reported cases at all.

The cases were 2.9% Oroya fever (A44.0), 13.1% verruga peruana (A44.1), and the rest (85.3%) were other forms of bartonelloses (A44.8-A44.9). The highest rates of Oroya fever were reported at Bolivar (2.5 cases/1,000,000 population) (Figure 2A).

For verruga peruana, the highest number of cases were reported in Antioquia (32; 17.8%; 5.21 cases/1,000,000 population) and the highest rate was at Magdalena (11.54 cases/1,000,000 population) (Risaralda, 6.45; Caldas, 5.1) (Figure 2B).

Other forms of bartonelloses were also reported at Magdalena (48.65 cases/1,000,000 population) (Huila, 32.8; La Guajira, 18.9). At Nariño, Putumayo, Amazonas, Cauca, and Valle del Cauca, 11.7% of the cases of the country were reported (Figure 2C).

Classifying by gender, there were 1,267 cases reported in women (5.43 cases/100,000 population) (Table 1), 121 cases were reported in men (0.53 cases/1,000,000 population), and one case was indefinite. By groups of ages, the range of 20 to 29 years presented the more cases (394 cases, 29% of the total) (Table 1).

**Table 1:** Distribution of cases by gender and age group

Variable	n	%
Sex		
Female	1267	91.2
Male	121	8.8
Age groups (years)		
0-9	53	3.82
10-19	118	8.50
20-29	394	28.37
30-39	363	26.13
40-49	256	18.43
50-59	124	8.93
60-69	30	2.16
70-79	34	2.45
80 and more	17	1.22

Cumulative rate of 5.06 cases/1,000,000 population) and range 30 to 39 years (363 cases, 26% of the total); cumulative rate of 5.77 cases/1,000,000 population) and range 40-49 years (256 cases, 18% of the total); and cumulative rate of 4.5 cases/1,000,000 population.

Regarding ethnic groups, the Black, mulattos, Afro-Colombians, and Afro-descendants ethnic groups reported 15 cases, the indigenous ethnic groups reported 10 cases, and the other ethnic groups reported 393 cases while in non-reported ethnic groups, 971 cases were reported of the total 1,389 cases.

For the social economic classification (SISBEN, *Sistema de Identificación de Potenciales Beneficiarios de Programas*, Identification System for Potential Program Beneficiaries, Colombia), level one (lowest and most vulnerable) reported 460 cases (33%), level two reported 75 cases (5%) while 682 and 172 were non-reported and non-applicant cases, respectively. For localities, municipalities reported 760 cases (55%), urban centers reported 74 cases (5%), and rural areas reported 113 cases (8%); 442 cases were non-defined (32%).

## Discussion

Bartonelloses are still neglected worldwide. Despite this, they are not included formally in the group of neglected tropical diseases. Research, assessment, surveillance, prevention, and control of these diseases is lacking. Although prevalent, they lack investigation. Apart from Peru, other countries in the region have not studied these diseases enough.

In Colombia, there are not enough studies on bartonelloses, particularly in the past decade. Unfortunately, there are only a few studies that have reported some cases, not even case reports. In this country, among the territories previously identified as endemic, Nariño Department has been historically considered the only one reporting such diseases, particularly Oroya fever. Nevertheless, we have found that at least 11 different departments of Colombia, including Nariño, have 16 species of *Lutzomyia *(Diptera: Psychodidae) that are able to transmit *Bartonella bacilliformis*, based on previous reports. Some areas, previously unknown for the presence of bartonelloses, have been also recently reported [3]. Just in Nariño, bartonelloses were considered a significant public health problem, which led to the promulgation of statements for the fight against it in 1940 [4]. In Nariño, *Lutzomyia* is also able to transmit *Leishmania*, particularly due to the suitability of eco-epidemiological conditions. Despite the limitations of this study, the number of cases and relevant incidence rates show that the relevance of bartonelloses in Colombia is higher than thought.

Bartonelloses can lead to severe disease, complications, and fatal outcomes. Some reports in the 1930s and later in different municipalities of Nariño such as Sandoná included a high number of cases (490) showing this clinical burden. Municipalities such as Consacá and Samaniego, in the same department, also reported cases as high as 249 and 149, respectively. Mortality due to bartonellosis was also reported in nine municipalities of that department, which accounted for 75% of all deaths. In those areas, in Southern Colombia, the entomological assessment revealed that three vector species were identified, with 683 specimens of *Lutzomyia columbiana*, six of *Lutzomyia osornoi, *and five of *Lutzomyia rosabali*[5].

In the north Caribbean coast, the Cordoba Department (mainly in rural areas) has been also the place for some related studies, which found a seroprevalence of 48.7% (39/80) (IgG), corresponding 77% to male gender and 23% to female. In those studies, *Bartonella quintana *was found in 45% (36/80) of subjects and *Bartonella henselae *in 30% (24/80). Even, coinfections between different *Bartonella *species were found; 21 of these individuals (26.2%) had antibodies to two species of *Bartonella* [5-6].

These tropical diseases have not only been studied from the perspective of the field of human medicine, but they have also been researched about in animal reservoirs in the Cordoba Department, from the veterinary medicine point of view, describing them as reemerging zoonoses [7].

The epidemiology of these pathogens has not been well-characterized in other countries of Latin America, but there are some reports that give a clue of how these pathogens are being distributed over the other countries. In Brazil, some studies tried to define their prevalence in blood donors from Campinas, Sao Paulo state. In a research assessing 500 subjects, authors showed that 16 of them were positive for *Bartonella *polymerase chain reaction (PCR) (3.2%). Furthermore, with immunoassays, they found positive antibodies for *B. henselae *and *B. quintana *in 16% and 32%, respectively. There was also evidence of a relationship between culture-negative endocarditis and *Bartonella *because 10 of 221 (4.5%; 95%CI 3.96%–5.09%) cases were PCR positive for *Bartonella*, two of them *B. quintana*, four *B. henselae,* and four *Bartonella *spp [8].

In other studies, for example, in Ecuador (1997), in the region of Manabí, there were 39 out of 213 people that were PCR positive for *Bartonella*. This study assessed relatives or neighbors of 11 index people who got the Peruvian verruga [10]. In Peru, surveillance of this disease has been enhanced in certain areas, particularly in 2015, following the report of 23 cases of acute Oroya’s fever. During the same year, there was also a record of the eruptive phase of this disease, including 33 cases that were confirmed, for a total of 66 cases and an incidence rate of 0.211 cases/100,000 population [11].

In our study, *B. bacilliformis *was found prevalent in Colombia at Nariño, Cauca, and Valle del Cauca departments but also at Antioquia, Caldas, Huila, La Guajira, and Risaralda, which were not previously considered endemic for this infection. In the three first departments, *Lutzomyia columbiana* has been incriminated as the potential vector, but published studies are not yet available in the other departments.

Given this distribution, transmission will be occurring, as seen in the reported cases. Also, bartonelloses are endemic in more areas than those previously described by classic reports of these diseases in the country.

Other studies about *Bartonella *infections in urban and rural dogs determined the prevalence of the *Bartonella *antibody in 455 domestic dogs from four tropical countries (Brazil, Colombia, Sri Lanka, and Vietnam) and detected *Bartonella *(deoxyribonucleic acid) DNA in a subset of these dogs, being important in as much as at least 30 *Bartonella *spp. and subspecies were identified in domestic animals, which would serve as major reservoirs of *Bartonella *spp. The results included 258 (56.7%) from Colombia (Bogota), with an antibody titers average of 256 for the three antigens: *B. henselae, B. clarridgeiae, *and *B. vinsonii *subspecies *berkhoffii *[12]. These results are consistent with our findings regarding distribution.

In addition to epidemiological studies, some case reports should also be considered in the assessment of the bartonelloses situation in Colombia. A case report of prolonged fever secondary to systemic cat scratch disease in Cali, Valle del Cauca, has been indicated as an unusual disease but with several clinical manifestations, mainly lymphadenopathy, in a pediatric patient with a clumsy clinical evolution [12]. In Medellin, Antioquia, the second-largest city in the country, a similar case report of a preschooler in a rural area. with an axillary mass and other lymph nodes in the neck and inguinal regions, with painless and spontaneous resolution, was also reported. In this case, the diagnosis of cat scratch disease was made and the patient was treated with antibiotics with a decrease in his symptomatology [13].

Again analyzing our results, this provides evidence that a majority of cases are reported in rural municipalities rather than in urban centers. These are areas with limited health care. Also, most of the cases were reported in young, working populations (73% distributed in 20-29-year-olds; 26% in 30-39-year-olds; and 18% at 40-49-year-olds). Also regarding gender, 91% of the total reported cases were in women while only 9% were in men, which should be analyzed in detail in further studies.

Regardless of the wide information provided by the RIPS, this should be carefully analyzed and interpreted, as this would be an underreporting of disease. In the future, the use of the new International Classification of Diseases, version 11 (ICD-11) should be necessary, with the advantage of having more disease codes and, in addition, species listed in the etiological agents section with their corresponding codes (Table 2).

**Table 2:** ICD-11 codes for bartonelloses (diseases and agents), World Health Organization, 2018 Obtained from the ICD-11 browser: https://icd.who.int/browse11/l-m/en ICD: International Classification of Diseases

ICD-11 Codes	
01 Certain infectious or parasitic diseases	
Other bacterial diseases	Diseases
1C11	Bartonellosis
1C11.0	Carrion disease
1C11.00	Oroya fever
1C11.01	Verruga peruana
1C11.1	Trench fever
1B98	Cat-scratch disease
1C11.Y	Other forms of bartonellosis
Extension codes	
Infectious Agents	
Bacteria	Agents
XN5PZ	Gram negative bacteria
XN3NJ	Bartonella
XN0W4	Bartonella bacilliformis
XN14D	Bartonella quintana
XN302	Bartonella koehlerae
XN3F6	Bartonella clarridgeiae
XN43H	Bartonella rochalimae
XN5J5	Bartonella elizabethae
XN5SH	Bartonella grahamii
XN6KD	Bartonella vinsonii
XN862	Bartonella henselae
XN94Y	Bartonella washoensis

The diagnosis of bartonelloses should be improved in its clinical suspicion as well as in laboratory tests available in the reported departments, where our study has found this pathogen to be prevalent. Bartonelloses should be included in the differential diagnoses of febrile syndrome, as well as in other common lymph node and skin manifestations in such endemic areas. This finally, would have not only public health implications but also for domestic and international travelers to those areas. Without a doubt, more research on bartonelloses is necessary for the country, including epidemiological studies on human, vector, and animal reservoirs of these bacterial infections.

## Conclusions

Infections due to *Bartonella bacilliformis* in Colombia were found distributed in Nariño, Cauca, and Valle del Cauca but also at Antioquia, Caldas, Huila, La Guajira, Risaralda, Cordoba, and the Caribbean area. Given this distribution, transmission would be occurring, as seen in reported cases, in more areas than previously described by classic reports of these diseases in the country. Further research is needed and expected in the near future, including seroprevalence surveys in the described areas, in areas where conditions for the vector are prevalent, and in areas of the country where the febrile syndrome in humans tends to be of unknown origin. Raising awareness with this and other studies is necessary in order to consider this and keep in mind the differential diagnoses.
